# Intermittent Fasting: Potential Bridge of Obesity and Diabetes to Health?

**DOI:** 10.3390/nu14050981

**Published:** 2022-02-25

**Authors:** Bo-Ying Zang, Li-Xia He, Ling Xue

**Affiliations:** 1 Department of Preventive Medicine, School of Publish Heath, North China University of Science and Technology, Tangshan 063000, China; zboying10@163.com; 2 Division of Molecular and Cellular Oncology, Dana-Farber Cancer Institute, Brigham and Women’s Hospital, Harvard Medical School, Boston, MA 02215, USA

**Keywords:** intermittent fasting, obesity, diabetes, weight loss, glucose, insulin

## Abstract

Obesity has been an escalating worldwide health problem for decades, and it is likely a risk factor of prediabetes and diabetes. Correlated with obesity, the number of diabetic patients is also remarkable. A modest weight loss (5–10%) is critical to alleviate the risk of any other metabolic disease. Reduced energy intake has been an essential factor for weight loss reduction. As a new behavior intervention to lose weight, intermittent fasting (IF) attracts considerable attention and has become a popular strategy among young people. IF is a diet pattern that cycles between periods of fasting and eating on a regular schedule, involving various types, mainly Intermittent Energy Restriction and Time-Restricted Fasting. Accumulating evidence shows that short-term IF has a greatly positive effect in animal studies and contributes favorable benefits in human trials as well. Nevertheless, as an emerging, diverse, and relatively premature behavior intervention, there are still limited studies considering patients with obesity and type 2 diabetes mellitus. It is also a controversial intervention for the treatment of metabolic disease and cancer. The risks and challenges appear consequently. Additionally, whether intermittent fasting can be applied to long-term clinical treatment, and whether it has side effects during the long-term period or not, demands more large-scale and long-term experiments.

## 1. Introduction

In recent decades, the global prevalence of obesity has increased significantly, from less than 1% in 1975 to 6–8% in 2016 [1]. Being overweight or obese causes at least 2.8 million people to die each year, which has also lead to an estimated 35.8 million (2.3%) global disability-adjusted life years (DALYs) [2]. Moreover, the incidence of diabetes in obesity patients is 3–4 times as high as that of the non-obese population. Increasing evidence indicates that overweight and obesity are related strongly to type 2 diabetes mellitus (T2DM) [3]. A large amount of cross-sectional and longitudinal studies indicate that body mass index (BMI) is positively related to the risk of T2DM. Many researchers believe that most cells of obese patients, especially fat cells, are insensitive to insulin, which thereafter leads to insulin resistance to some extent [3]. The prevention and treatment of obesity has necessitated a global health strategy to control the escalating epidemic of chronic diseases caused by obesity, including diabetes.

As an attractive field, intermittent fasting (IF) is currently one of the most popular ways to control caloric intake, leading to weight loss and improved health and lifestyles. IF is a dietary pattern that cycles between periods of fasting and regular eating on a regular schedule, during which the subjects severely restrict their calorie intake or consume no food. As a special form of caloric restriction (CR), it also induces a long-term effect—“metabolic adaptation”, which can reduce the metabolic rate and may prolong human lifespan [4]. Plenty of animal and clinical studies have demonstrated that IF benefits human health in many ways, such as losing weight [5], preventing metabolic diseases [6], and improving glucose tolerance [7] and muscle function [8]. The positive effects on weight loss by IF have been studied, including trials of both humans and animals. Grosso [9] once pointed out that these findings may be explained by the fact that, as IF is embedded in the lifestyle, the moderate physical activity and energy intake are balanced in a long-term way. Moreover, many other diets already exist for clinical intervention, religious customs, and other purposes, showing similar effects to IF, such as the Mediterranean Diet, Ramadan Intermittent Fasting, Christian Orthodox Fasting (OF), and so on. Of note, according to recent advances, OF may have a positive metabolic effect that is related to lower lipid concentrations [10] and improved adiponectin concentrations [11].

Epidemiological evidence proving the advantages of IF is also gradually emerging. In a cross-sectional study, TRF-8 (feeding within 8 h) and TRF-10 (feeding time within 10 h) were conducted on 1936 adults, and Currenti et al. [12] found that TRF and being overweight with hypertension showed an inverse correlation, and TRF-10 was also inversely associated with dyslipidemias.

Meanwhile, negative effects also have been found to accompany IF. Corley et al. [13] have pointed out the rising risk of hypoglycemia with IF when implemented in T2DM patients. There are also different opinions regarding other effects brought about by IF, such as whether the variation of gut microbiota occurs or not after fasting, which requires more studies. Whether the effect of IF only relates to the caloric restriction or acts as a lifestyle is still under confirmation. In this article, we will review the beneficial and potential harmful effects of IF on health, and discuss whether IF is suitable as a behavioral intervention treatment for obese, diabetic patients and potential sub-healthy people from different aspects.

## 2. Intermittent Fasting and Its Common Forms

IF is implemented to lose weight and reduce health hazards caused by obesity. In recent studies, there are more and more different viewpoints about the level of weight loss mediated by IF. Meanwhile, other effects such as glycemia regulation have been proposed in recent years. Compared with traditional means of losing weight, IF concentrates both on specific mealtimes and energy intakes instead of energy intake restriction uniquely. Diet and diet interventions with sports are basic methods to reduce weight. Temporal rhythms were considered when time-restricted fasting was conducted. As an emerging unique weight loss method, the commonly known intermittent fasting has two major forms: Intermittent Energy Restriction (IER) and Time-Restricted Fasting (TRF).

Intermittent Energy Restriction (IER), also known as periodic fasting (PF), is a more common pattern of IF, which involves short periods of substantial energy restriction (75–100%) alternated with a normal diet [14]. IER mainly promotes weight loss by decreasing energy intake. It involves a period of fasting days followed by a period of feeding days. The regimens of IER act as an alternating set of days, such as 5 fasting days followed by 2 feeding days, which can also be called a twice-weekly fast [15]. Alternative Day Fasting (ADF), as a special form of IER, is conducted within a fixed period as one fasting day and one feeding day alternately. In general, the cycle time of IER is no more than one week. Compared with the traditional daily energy restriction (DER, a diet of fasting on all days), which is also called continuous energy restriction, in which energy restriction is carried out every day, IER reduces compensatory metabolic responses, such as the compensatory reduction of “resting energy expenditure” [16]. The previous study showed that IER increases compliance without compromising the weight loss effect [17]. IER is an ideal alternative strategy to DER according to the current study’s results.

Time-Restricted Fasting (TRF) is a daily fasting pattern that arose within the context of circadian rhythms, which are led by daily changes with alternating light and dark. Most species store energy in the daylight when food is available (the feeding period) to utilize during the rest of the day (the fasting period), without impairing fitness and vitality. The alternating cycle of feeding and fasting is called ‘‘fasting physiology’’. Fasting physiology is triggered earlier as feeding time is restricted, thereby achieving the purpose of increasing energy consumption [15]. Meanwhile, the whole-body metabolism is deeply impacted by circadian rhythms in the animal itself, which has been reported by recent studies. Lundell et al. [18] have confirmed that TRF improves metabolism through altering lipid and amino acid metabolite rhythmicity, without perturbing clock gene expression. Most species alternate the fasting period coinciding with sleep. TRF requires all energy intakes to be finished within certain hours per day, usually limited to 8–10 h or less [19]. The most common form is 16:8, in which all energy intakes are controlled in 8 h and the fasting is performed during the remaining 16 h [20]. Furthermore, the 15:9 diet also frequently appears in recent studies, in which all energy intakes are controlled in 15 h and the fasting is performed in the remaining 9 h. The difference between TRF and IER is shown in Table 1.

**Figure 1:**
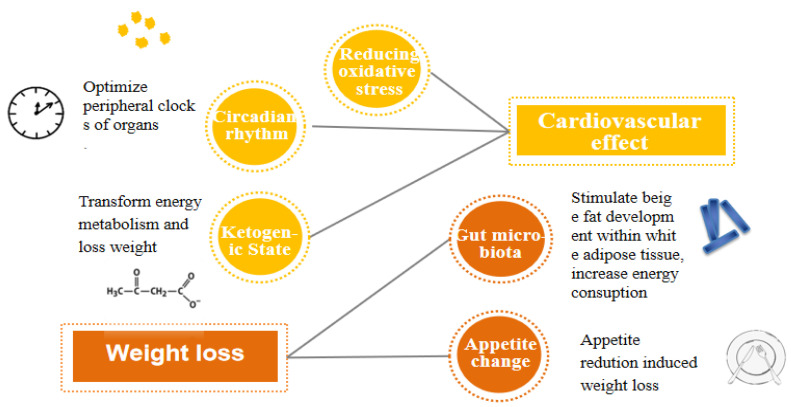
The possible mechanisms of intermittent fasting that influence metabolism.

The underlying mechanisms of how IF influences metabolism still remain unclear. Some potential relevant mechanisms are proposed noticeably regarding cardiovascular aspects and weight loss. Reducing oxidative stress, circadian rhythms, and the ketogenic state are the three major hypotheses about how IF leads to cardiovascular benefits [21]. Alternatively, the testified mechanism of weight loss led by IF is concluded as two relevant aspects: gut microbiota and appetite change (Figure 1).

**Figure 2:**
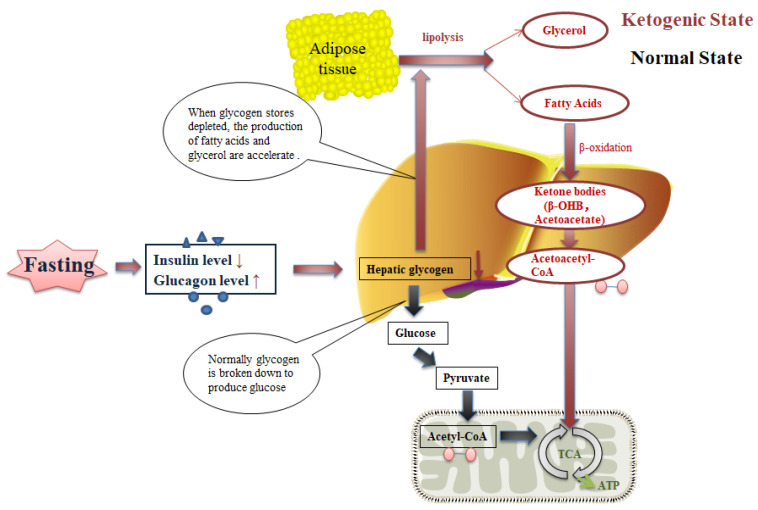
Two states of energy supply before and after long-term fasting. The lipids in adipose tissue are metabolized to free fatty acids and glycerol, which are released in blood and transported into hepatocytes, and then metabolized by β-oxidation to produce the ketones β-hydroxybutyrate (β-OHB) and acetoacetate.

The oxidative stress hypothesis supports the notion that calorie restriction hinders mitochondria from producing more free radicals and lowers both systemic and vascular inflammation in obese patients, thus improving the symptoms and pulmonary function of obese asthma patients [21]. Focusing on a different aspect, the circadian rhythm hypothesis indicates that fasting properly optimizes the peripheral clocks of organs, which can be embodied by TRF through restricting feeding times to a specific time period. Accumulating evidence demonstrates that the final outcome varies from different feeding time periods. A randomized crossover trial study showed that earlier feeding time period restriction reduced mean fasting glucose [22]. Additionally, the ketogenic state hypothesis suggests that IF induces a ketogenic state, where more energy is required by ketones, thus promoting weight loss [21]. During the initial post-absorptive phase (0–3 h after cessation of food consumption), the insulin level starts to fall, and the glucagon level rises, thereafter promoting the breakdown of the liver glycogen store, which lasts 6–24 h typically [23]. When the glycogen stores in hepatocytes are depleted, adipose tissue lipolysis is accelerated to produce more glycerol and fatty acids. Gradually, the body’s preferential energy utilization shifts from glucose by glycogenolysis to fatty acids and fatty acid-derived ketones, usually occurring between 12 and 36 h after energy intake cessation [24] (Figure 2). Ketones are transported to muscle cells and neurons, in which adenosine triphosphate (ATP) is generated after being metabolized to acetyl coenzyme.

Besides energy restriction, there are also different theories about how IF could give rise to weight loss. It has been revealed that the every-other-day fasting (EODF, feed every other day, also called alternate-day fasting) regimen significantly ameliorates obesity by selectively stimulating beige fat development in white adipose tissue (WAT). In an in vivo study using C57BL/6N mice, Li et al. [25] showed that EODF did not affect cumulative food intake, thus suggesting that weight loss might be induced by the increased energy expenditure. They proposed that this phenomenon is probably induced by the reshaping of the gut microbiota, which leads to the elevation of fermentation to produce acetate and lactate and, subsequently, to the induction of WAT beiging. Moreover, in a human randomized study of TRF, it was verified that the influence of weight loss was primarily induced by appetite reduction and increased fat oxidation rather than by energy expenditure elevation [26]. The occurrence of these two diametrically opposite conclusions may result from the different forms of IF intervention. There are also some other mechanisms by which IF may affect metabolism, such as glucose–insulin metabolism, muscle function, and cognitive function, which will be reviewed in the next section.

The growing evidence suggests that the timing of the fasting is a key factor, which makes IF a more realistic, sustainable, and effective approach for the prevention of diabetes and obesity. However, consequent skepticism has emerged over the adverse effects, such as the appearance of hypoglycemia, which also deserves to be probed.

## 3. Intermittent Fasting and Obesity

Obesity is a worldwide health problem that has been defined as a disease by WHO, and continues to be a major focus of public health efforts worldwide [27]. Obesity is mostly caused by an excess fat ratio and subsequently induces many other metabolic diseases. The complications mainly include hypertension, hyperlipidemia, diabetes mellitus, coronary heart disease, and stroke. Fitzgerald et al. [28] concluded that the consequences can be divided into three categories: the increased mechanical burden of obesity; the dangers of increased food intake, or of the “wrong sort” of food; and the miscellaneous complications and associations of obesity. The increased mechanical burden mainly includes accelerating the degenerative joint diseases, respiratory compromise, and cardiomegaly. Hypertension and diabetes mellitus, as two of the most important miscellaneous complication, are the major focus in obesity. Obesity can also lead to obesity stigma, which threatens health, generates health disparities, and interferes with effective obesity intervention efforts, posing numerous consequences for patients’ psychological and physical health. Obesity stigma is universal and unmarked to the public and becomes an invisible harm to obese patients [29].

IF appears as a nascent effective method that may be very beneficial to obesity. There are some animal studies and several human trials showing the encouraging effects of IF on obesity. Alongside the main benefit of reducing weight gain, IF also affects visceral obesity and reduces the cardiometabolic risk [30], regulates metabolism [31], recovers the muscle function [32], and improves glucose homeostasis [33].

### 3.1. Body Weight

An important manifestation of obesity is an excessive body weight, while the degree of weight loss induced by treatment varies from different studies. Importantly and intriguingly, the two diet patterns of IF, IER and TRF, have been concluded to promote weight loss in both animal studies and human clinical trials. The accumulating animal studies showed that IF could reduce weight gain in a diet-induced obesity mice model. Kim et al. [34] observed that a 2:1 isocaloric IF (comprising 2 feeding days followed by 1 fasting day) regimen caused the substantial induction of weight loss among hyperphagic ob/ob mice treated with 2:1 IF, while showing a 21% caloric intake reduction, compared to ob/ob ad libitum mice. More importantly, the weight loss did not affect lean mass and specifically reduced fat mass according to the composition analysis, suggesting that isocaloric IF potentially targeted the reduction of body weight via eliminating fat mass without destroying muscles. The underlying mechanisms driving the selective source of energy expenditure are speculated to be related to the modulation of autophagy and the gut microbiome, which has been elucidated in many recent studies. Consistently, Olsen et al. [35] testified to the effect of TRF on weight loss in rats, and also proved that TRF can restrict weight increase in obese rats. Additionally, this study demonstrated that high-fat-diet-induced weight gain in juvenile rats was suppressed without reducing total calorie intake when they were treated with a TRF regimen with 9 h interval restricting feeding during the dark phase on 5 weekdays per week. Although the perturbation of the circadian rhythm of eating behavior is considered a critical contributor to the elevated energy expenditure, the improvement of a lower oxidative capacity in the muscles of diet-induced obese rats by TRF treatment, meaning that more fat is being used for energy expenditure, would be a more directly involved mechanism. Supporting the animal studies, the beneficial effect of IF on weight loss has been also investigated in clinical trials. Catenacci et al. [5] reported that the weight of obese adults dropped by 8.2 ± 0.9 kg after an 8-week intervention of zero-calorie ADF with one fasting day (0 calorie needs) and one feeding day alternating. It is worth noting that the 8-week zero-calorie ADF has been verified to be safe and tolerable by this study.

Nevertheless, it is worth noting that isocaloric IF may not be an effective approach for regulating body weight in genetical obesity when compared to diet-induced obesity. Minimum weight loss was observed on genetically obese mice and wild-type (control) mice after 18 days of ADF, consisting of ad libitum chow availability for 24 h followed by complete fasting for the next 24 h [36]. For wild-type mice, however, this ADF protocol did not appear to influence total food intake either, as the mice on the ADF regimen consumed double the amount of food of control mice on the ad libitum regimen on feeding days, potentially resulting in the absence of weight loss with the ADF protocol. Conversely, ADF leptin-deficient ob/ob mice consumed 37% less food than ad libitum ob/ob mice; consequently, the blunted weight loss was likely attributed to the lower metabolic rate after the induction of torpor on fasted days. Kim et al. [37] found that isocaloric IF failed to induce caloric-independent weight loss in ob/ob mice after 16 weeks of 2:1 IF (2 days of feeding followed by 1 day of fasting) treatment. It was presumed that this phenomenon was possibly associated with the three following major contributing factors: leptin deficiency and the hyperphagic nature of ob/ob mice; insufficient induction of fasting-mediated adipose-VEGF expression, which is critical for WAT browning; and the abnormal microbiome composition and the altered population of microbiota, which exert a pivotal role in energy metabolism and beige adipogenesis. As a key adipokine in energy homeostasis and thermogenic activity, the expression of leptin is markedly regulated by fasting and feeding. In particular, ob/ob mice develop severe fasting-induced hypothermia, along with hyperphagia, which thereafter lead to excessive calorie intake directly. Intriguingly, this could be suppressed by IF treatment to avoid extra intake. Leptin also impacts hypothalamic pro-opiomelanocortin (POMC) neurons in the promotion of adipose thermogenesis, including WAT browning. It has been demonstrated that leptin is an upstream regulator of VEGF expression in endothelial and cancer cells [38,39,40]; accordingly, insufficient fasting-induced adipose-VEGF induction by leptin deficiency may undermine the WAT browning induced by IF in ob/ob mice [37]. Furthermore, it has been revealed that microbiota metabolites contribute to the IF-induced beige adipogenesis in WAT [25]. Therefore, it is reasonable to speculate that the dysbiotic gut microbiota in ob/ob mice may be related to reduced energy expenditure and the perturbed browning capacity of the WAT. Moreover, due to the limited long-term evidence, the benefit of weight loss may potentially rely on strict energy restriction. More large-scale cohort studies need to be performed to clarify the long-term benefits and potential harms amongst patients.

### 3.2. Abdominal Obesity, Visceral Obesity, and Cardiometabolic Risk

The 2003 Guidelines for Prevention and Control of Overweight and Obesity in Chinese Adults pointed out that, among overweight people, a male waist circumference greater than 90 cm and a female waist circumference greater than 85 cm, independent of BMI, are diagnosed as abdominal obesity. Abdominal obesity is one of the most important risk factors of hypertension, diabetes, and other cardiovascular diseases. Visceral obesity is caused by body fat deposition in the upper part of the body and within the abdominal cavity [41]. Visceral adipose tissue is located near vital organs, such as the liver, stomach, and intestines, and even builds up in the arteries. Visceral obesity can actively increase the risk of adverse health problems, such as causing fatty liver and disturbing metabolism, and eventually may lead to cardiometabolic disorder or diabetes.

Importantly, both TRF and ADF have been reported to effectively reduce abdominal obesity through multiple human trials. A clinical trial carried out on abdominal obese participants showed that the mean waist circumference of 40 participants was shortened by 5.3 ± 3.1 cm after 3 months of TRF intervention [42]. Likewise, it has also been proven that ADF it is an efficient and applicable strategy to reduce abdominal obesity in abdominal obese patients. For instant, Park et al. [30] conducted a meta-analysis recently indicating that ADF reduced the body mass index, body weight, waist circumference, and total cholesterol in overweight and obese adults. This meta-analysis showed that waist circumference was dramatically affected by ADF, which decreased by 4.00 cm (weighted mean difference). Of note, it was suggested to be only applicable to obese adults aged 40 years or older. With the reduction of the abdominal circumference, thereafter, IF can be potentially beneficial for decreasing the cardiometabolic risk and the risk of other metabolic diseases, and further long-term, large-scale population trials remain to be carried out.

### 3.3. Metabolism

The effect of IF on metabolism mainly focuses on glucose and lipid homeostasis, which specifically manifest in improvements in insulin sensitivity and glucose tolerance. Kim et al. [37] reported that Ob-IF (subjected IF) mice showed smaller glucose excursions than Ob-PF (subjected paired feed al libitum) mice in a glucose tolerance test (GTT), suggesting that glucose homeostasis was improved in ob/ob mice subjected to IF. Whereas this phenomenon does not originate from the difference in body weight and insulin sensitivity, as a substitute, IF significantly improved glucose handling capacity via promoting insulin secretion as the action of GLP-1 in glucose increase. Consistently, Liu et al. [31] also found that the fat mass and glucose tolerance was improved in their 8-week IF treatment study on high-fat-diet-fed mice. The therapeutical benefits of IF on metabolism improvement have been also proven in clinical trials. For instance, Antoni et al. [14] suggested that IER acutely altered postprandial glucose in a three-day randomized crossover trial on ten overweight/obese participants. Compared with the control group, total ER (100% restriction) led to a significant reduction in fasting glucose, and glucose iAUC in response to the liquid test meal was significantly greater. As for the potential mechanism, Hatori et al. [6] found that the metabolic disease benefit of TRF is mainly associated with the temporal reprogramming of glucose metabolism away from gluconeogenesis towards glycolysis, reducing glutathione and anabolic pathways [6]. The glucose homeostasis and anabolic metabolism in liver are coordinated among circadian oscillator components and metabolic regulators, and also a large number of downstream effectors. As such, the reduced dephosphorylation of glucose-6-phosphate contributes to a decrease in the release of free glucose and subsequently lowers blood glucose. Thus, the improved glucose metabolism by IF will be a potentially favorable benefit for obesity management.

In addition to glucose metabolism, IF also plays an important role in controlling lipometabolism. A randomized, controlled, long-term clinical trial performed with the 5:2 approach to IER showed that the metabolic indexes were improved in participants with abdominal obesity, such as lipids and glycemia, indicating a lower cardiometabolic risk after a one-year intervention [43]. Nonetheless, it was revealed that a short-term TRF (fasting for 8 h during the inactive/light phase for 4 days) led to hepatic steatosis and temporarily promoted hepatic lipid accumulation and lipogenesis in mice with a high-fat diet, as the mRNA expression of enzymes involved in lipogenesis was increased [44]. Importantly, the increased triglyceride accumulation may be lost over time since accumulating evidence demonstrates diminished hepatic triglycerides in mice with a much longer timeframe of IF [6,45,46,47].

Notably, the IER intervention approach to weight loss generally involves short periods of substantial energy restriction (>70%) interspersed with a normal diet. Antoni et al. [14] reported that a day (36 h) of substantial energy restriction can acutely alter glucose–lipid metabolism, as well as incomplete energy intake compensation among overweight and obese participants, with partial energy restriction (75%) producing the more favorable overall response. They presumed that acute disturbances in fuel management could lead to changes in metabolic flexibility and even cardiometabolic risk. This suggests that IF would be harmful to health when carried out unqualified. Overall, existing evidence both in animal studies and human trials demonstrates that an appropriate IF intervention has a potentially favorable effect on regulating metabolism in obesity.

Further investigation is required to reveal how the metabolism reconciles over time to the repeated perturbations through different types of IF, as well as to explore the implications of IF for long-term health.

### 3.4. Muscle Function

There can be substantial heterogeneity of muscle fiber types in humans. Obesity has been verified to affect the contractile function of isolated skeletal muscle [8]. It is reported that obesity induces a shift in the fiber types of muscle from slow to fast by disrupting calcium signaling and 5′-adenosine monophosphate activated protein kinase (AMPK) activity, subsequently promoting the class II histone deacetylase (HDAC)-mediated inhibition of the myocyte enhancer factor 2 (MEF2), which promotes slow fiber type expression and causes a series of damage, such as exercise intolerance and changes in muscle function. Notably, it has been testified that TRF restores muscle function in animal studies. For instance, Villanueva et al. [32] observed mitochondrial abnormalities and sarcomere disorganization in obese Drosophila models induced by a 3-week high-fat diet (HFD). However, TRF increased the HFD flight performance and suppressed skeletal muscle dysfunction and intramuscular lipid infiltration produced by circadian light disruptions, while a HFD ad libitum accelerated the decrease in functional performance and locomotion under equal food consumption, which indicated that TRF restored muscle dysfunction, which was mediated by circadian rhythms.

Obesity-induced attenuation of calcium signaling, mediated by calcineurin, adiponectin, and actinin, affects the excitation–contraction coupling and excitation–transcription coupling in the myocyte, and eventually impacts in vivo and in vitro muscle mobility. Moreover, adiponectin reduction induced by obesity also decreases insulin sensitivity, although it increases the circulating insulin level, which inhibits AMPK activity in muscles and thereby slows down fiber-type expression. This phenomenon can be reversed by TRF according to a recent study. In a clinical trial, 16 healthy males were assigned to two groups that underwent either 2 weeks of eTRF (energy intake restricted to between 08:00 and 16:00) or a control/caloric restriction diet [48]. The result demonstrated that eTRF improved whole-body insulin sensitivity and glucose uptake in skeletal muscle. 

Therefore, the current evidence has proven the efficacy of IF in the improvement of muscle function in obesity patients, which probably is involved in attenuating the impaired calcium signaling pathway or/and glucose uptake caused by insulin insensitivity.

### 3.5. Hippocampus-Dependent Cognition

Adult neurogenesis occurs in the subventricular zone of dentate gyrus in human hippocampal formation, which is the generation of new neurons throughout life [33]. Increasing evidence suggests that different diets can alter the brain structure and function. To explore the effects of intermittent and continuous energy restriction on human hippocampal neurogenesis-related cognition, a four-week randomized controlled human trial was performed in an adult human population with central obesity. The data indicated that although no significant difference was noted in cognitive improvement between the two types of energy restriction diets, a significant improvement appeared in separation performance under both energy restriction diets when analyzed as a single cohort [33]. Implicit in this finding is that any form of energy restriction might mediate memory function, possibly via modulating human adult hippocampal neurogenesis. This discovery may offer new insights into the IF treatment and the prevention of memory decline and provides a novel therapeutic approach for the behavior intervention of cognitive disease. However, as there is no placebo control group to compare for the pattern separation task improvement, more trials are needed to verify the accuracy of this conclusion.

### 3.6. Gut Microbiota

*Firmicutes* and *Bacteroidetes* are two major phyla that are dominant in the human gut microbiota. It is noted that there is a symbiotic relationship between *Firmicutes* and *Bacteroidetes*, which promotes the host to absorb or store energy together. Therefore, the *Firmicutes* to *Bacteroidetes* ratio (F/B ratio) has been extensively analyzed to evaluate its important influence in maintaining intestinal microbiome homeostasis in humans and mice [49]. Previous studies demonstrated that obese animals and humans exhibit a higher *Firmicutes/Bacteroidetes* ratio compared with normal-weight individuals; importantly, fasting and feeding rhythms have an influence on shaping the gut microbiota [50]. In a study that explored whether every-other-day fasting (EODF) could selectively activate beige fat thermogenesis via a microbiota–beige fat axis, it was indicated that the abundance of the gut microbiome in obesity is potentially changed with IF intervention. A significant difference in Operational Taxonomic Unit (OTU) abundance of *Firmicutes* and *Bacteroidetes* on the phylum level was observed between the AL mice (fed on ad libitum regimen) and EODF mice (fed on every-other-day fasting regimen) in 15 cycles of ADF treatment (fed with alternating 24 h periods of free access to food followed by 24 h fasting) [25]. The ratio of *Firmicutes/Bacteroidetes* was increased from 3.4 in AL mice to 8.9 in EODF mice. The shift was inferred to be associated with the increased glucose uptake in inguinal white adipose tissue (WAT). Intriguingly, and in contrast, to investigate the effects of IF on changes in gut microbiota in obesity, Gabel et al. [51] conducted a 12-week clinical trial among obesity patients treated with 16:8 diet TRF, and then 16S rRNA gene sequencing in the stool samples was performed to further determine whether or not the gut microbiota were affected by TRF. Unfortunately, the abundance of *Firmicutes* and *Bacteroidetes* and the diversity of the gut microbiome were not found to display significant changes after 12 weeks of TRF. The reason for the inconsistent results was assumed to be that the limit weight reduction (2%) and energy restriction (20%) produced by the present TRF intervention were not enough to alter the composition of the gut microbiome beneficially. The effects of IF on obesity in animal studies and clinical trials are summarized in Table 2 and Table 3.

## 4. Intermittent Fasting and Type 2 Diabetes Mellitus (T2DM)

Obese patients are 3–4 times more likely to develop diabetes than non-obese patients. As the most common type of diabetes, T2DM accounts for 90% of diabetic patients. Its pathogenesis is involved in defective insulin secretion by pancreatic β-cells and the inability of insulin-sensitive tissues to respond appropriately to insulin, which means that patients have to rely on external insulin [52]. Clinical treatment has been committed to improving insulin sensitivity, wherein IF also is claimed to play a favorable role in T2DM. Accumulating evidence has verified the beneficial effects of IF on both diabetic animals and T2DM patients. In the meantime, it should be noted that the impact of IF is riskier on diabetes patients than simply obese patients.

### 4.1. Weight Loss

As many obese patients have a risk of developing diabetes, the weight loss benefits of IF can be very helpful for patients with diabetes, especially T2DM. Importantly, consistent improvements in weight loss have been noted with different patterns of IF intervention. A 12-month intervention with IF (2100–2500 kJ diet 2 non-consecutive days/week and their usual diet for 5 days/week) in a clinical trial with T2DM patients showed that weight loss was maintained at −3.9 kg at 24 months [55]. Similarly, in a large-scale human trial, wherein 741 subjects were included and 697 completed, it was shown that the weight reductions in both the T2DM group and no diabetes group triggered by periodic fasting therapy (an Expert Panel Update of the 2002 Consensus Guidelines) [56], respectively, were −5.29 ± 2.56 kg and −4.31 ± 2.41 kg [50]. Together with the accumulating evidence, it is worth noting that IF efficacy for weight loss in diabetes is more consistent and reliable when the intervention period is appropriate and the design is more feasible. Similarly, because of the small sample size in most trials, long-term studies are needed to explore whether the weight loss may be directly caused by the intervention of energy limitation during the fasting period or not.

### 4.2. Metabolism

Along the lines of obesity, the effect of IF on T2DM patients majorly involves the modulation of lipometabolism and glucose metabolism. TRF (15:9 diet) has been demonstrated to improve glucose tolerance. A remarkable reduction in glucose incremental area under the curve was observed in a randomized crossover clinical trial including 15 male adults at risk for type 2 diabetes [22]. In the same study, the authors also reported that only early TRF (TRFe, feeding time from 8 a.m. to 5 p.m.) decreased mean fasting glucose, but not delayed TRF (TRFd, feeding time from 12 p.m. to 9 p.m.). Likewise, Liu et al. [7] indicated that ADF could restore the autophagic flux in islets induced through continuously consuming high-fat food, and it could improve glucose tolerance by enhancing glucose-stimulated insulin secretion, beta cell survival, and the nuclear expression of NEUROG3, an important marker of pancreatic regeneration in obesity-induced diabetes.

As a metabolic disease, diabetes is generally companied by other metabolic disorders such as non-alcoholic fatty liver disease. To determine whether or not IF influences metabolic disorder in diabetes, Drinda et al. [57] carried out a prospective observation clinical study about the effects and safety of periodic fasting in subjects with and without T2DM. The binary logistic regression results indicated that every day of fasting increased by 40% the chance of improving a manifest fatty liver (fat liver index > 60) and shifted to a lower category of risk; moreover, after fasting, nearly half of the 264 subjects in the high-risk category switched to the low-risk category, suggesting that periodic fasting dramatically decreased the risk of non-alcoholic fatty liver disease in subjects both with and without T2DM.

The potential uncertainty of performing IF treatment in T2DM patients should be considered and the side effects should be well noted. IF has been reported to increase the risk of hypoglycemia in T2DM patients who are treated with hypoglycemic medications. Not surprisingly, the amount of hypoglycemia cases alters with the degree of medication. For instant, Corley et al. [13] reported that the risk of having a hypoglycemic event was twofold greater during fasting despite medication reduction. Nevertheless, they also indicated that the risk of hypoglycemia appeared to be more likely related to individual characteristics than patterns of fasting, and the hypoglycemia events were also relatively low and in an acceptable rate.

### 4.3. Gut Microbiota

There is a strong correlation between the diversity of the gut microbiota and the existence of diabetes [3]. Gut microbiota composition was altered significantly in people with impaired glucose tolerance and combined glucose intolerance and T2DM [58]. This observation is supported by other studies as well. A 7-month animal study conducted with ADF (fasting for 24 h every other day) collected fecal samples and performed genomic DNA sequencing, and the data indicated that the gut microbiota composition might be altered by long-term IF, wherein the represented phyla were *Bacteroidetes, Firmicutes, Verrucomicrobia, Tenericutes*, *Actinobacteria*, and *Proteobacteria* [59]. Beli et al. [59] inferred that the alternation in the microflora in db/db mice with the IF regimen could promote the integrity of the gut barrier and eliminate the risk of diseases caused by the disruption of the gut barrier. The diversity of the gut microbiota is also associated with energy metabolism and metabolic signaling. Consistent with the known modulatory effects of *Firmicutes* on bile acid metabolism, a significant increase in a neuroprotective bile acid, tauroursodeoxycholate (TUDCA), was observed in db/db mice on IF, and subsequently the receptor of TUDCA, TGR5, mediating TUDCA’s effects in the retina, was activated for retinal protection to prevent the development of diabetic retinopathy. No improvement in glucose control was noted in the absence of weight loss.

### 4.4. Lifespan

A healthy diet style impacts lifespan, and modulating the diet and the application of fasting patterns are new lifestyle approaches that lead to an increased lifespan. In a 7-month study, it was indicated that IF feeding prolonged the lifetime of db/db mice in the absence of improving glycemic control [59]. The survival rate of db/db mice with the ADF regimen was significantly higher than those on an ad libitum feeding regimen; however, no change in the glycated hemoglobin level was observed. Additionally, diabetic retinopathy was observed in db/db mice in this study, and interestingly, IF also inhibited the development of acellular capillaries and the infiltration of inflammatory cells in the retinas of db/db mice. Therefore, it is reasonable to speculate that IF could be protective against renal histological injury to prolong the lifespan of db/db mice.

**Figure 3:**
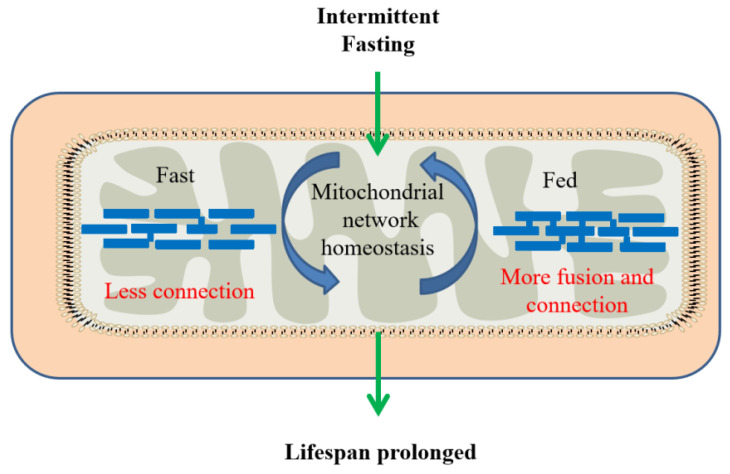
The interaction between intermittent fasting and mitochondrial network homeostasis.

Although this result is intriguing, it is more important to understand the physiological role of mitochondrial network remodeling in longevity. It is noted that maintaining mitochondrial network homeostasis is sufficient to extend the lifespan and mitochondrial network plasticity is required for IF-mediated longevity. It was proposed that AMPK and diet restriction promoted longevity by remodeling and maintaining mitochondrial network homeostasis and functional coordination with peroxisome to increase fatty acid oxidation [60]. IF-mediated longevity is involved in the dynamic remodeling of the mitochondrial network by fusion and fission in response to changing nutrient availability between fasting and feeding periods to protect from age-related changes and improve the lifespan (Figure 3).

Taken together, the substantially favorable influences of IF on body weight, metabolism, the abundance of gut microbiota, and lifespan have been observed in animal studies and human trials. Whether or not side effects appear with long-term implementation needs further investigation, in order to confirm the security and effectiveness of IF. The influence of IF in older patients and children and its underlying mechanism also remain to be further explored. The effects of IF on diabetes are summarized in Table 4.

## 5. Comparisons to Other Regimens

Different from traditional fasting, such as patients given nothing to eat, intermittent fasting sometimes demonstrates superiority in response to challenges. Some studies point out that the effect of an IF diet on body metabolism is similar to a traditional diet; however, IF requires higher compliance. Fortunately, most studies also showed a high degree of completion compared with traditional diets. In addition to the compliance, the efficacy of IF on influencing body composition and metabolism has been compared to other regimens, such as Daily Calorie Restriction, the Mediterranean Diet and Paleolithic Diet, the Ketogenic Diet, Ramadan Intermittent Fasting, and Christian Orthodox Fasting.

### 5.1. Comparison to Daily Calorie Restriction

Daily Calorie Restriction is a traditional diet to lose weight that requires a reduction in daily energy needs by 20–30%. Gabel et al. [61] conducted a study that compared the effects of ADF with those of Daily Calorie Restriction (CR) on body weight and glucoregulatory factors in overweight and obese adults with insulin resistance. The study lasted for 12 months and 43 participants with insulin resistance were tested. The results showed that there was no significant difference in weight loss between ADF and CR, but ADF caused sharper decreases in fasting insulin and insulin resistance in contrast to CR, indicating that ADF probably demonstrates a stronger influence on the intervention of type 2 diabetes via the induction of insulin tolerance. Smith et al. [62] compared ADF, TRF, CR, the Daniel Fast, and a high-fat western diet by assessing the differences in body composition, insulin, fasting glucose, and glucose tolerance test area under the glucose curve (AUC). This revealed that the fasting glucose and glucose tolerance under ADF were better than those under CR, although the other indicators were similar between ADF and CR, including the weight loss and fat mass gain.

### 5.2. Comparison to Mediterranean Diet and Paleolithic Diet

The Mediterranean Diet is characterized by a high intake of vegetables, legumes, fresh fruit, non-refined cereals, nuts, and olive oil, accompanied by moderate consumption of fish and dairy, a low intake of red meats, and moderate use of ethanol, mainly red wine consumed during the main meal [63]. The Paleolithic Diet (Paleo Diet) seeks to maintain the eating habits of the hunter–gatherer by restricting grains, legumes, and dairy to improve health. However, the concept conflicts with the accumulating evidence regarding their benefits for chronic disease prevention, which was observed in 58 clinical trials including 4635 adults in a meta-analysis [64]. In a 12-month randomized controlled trial, a total of 250 overweight (BMI (in kg/m^2^) ≥ 27) healthy adults were allowed to choose one diet from among the Paleolithic Diet, IF, and Mediterranean Diet [65]. It was found that most participants chose ADF to help the most with losing weight, and the Paleo Diet was chosen by the fewest participants to help the least with losing weight. The systolic blood pressure was decreased relatively more with the Mediterranean Diet. There were no significant differences in the other metabolic indices among the diets chosen by participants. These findings suggested that IF and the Mediterranean Diet can be effective for weight loss, and any of these dietary approaches may positively influence health, but the conflict of the Paleo Diet with grain benefits needs more attention and investigation.

Moreover, the Mediterranean Diet was proven to be associated with a significant improvement in health status with more sufficient evidence [66,67]. According to a meta-analysis of prospective cohort studies [67], the Mediterranean Diet can significantly decrease the risk of overall mortality, mortality from cardiovascular diseases and cancer, and the incidence of cancer, Parkinson’s disease, and Alzheimer’s disease. While further long-term evidence emerges, the Mediterranean Diet is still encouraged more frequently than IF.

### 5.3. Comparison to Ketogenic Diet

The Ketogenic Diet is a high-fat, low-carbohydrate diet that replicates the effects of fasting, and beneficial effects have been ascribed to the production of ketones, and it has been used for losing weight and suppressing seizures in epilepsy patients [68]. The very low-carbohydrate Ketogenic Diet promotes a shift in energy metabolism from carbohydrates to triglycerides, with the formation of ketone bodies (i.e., β-hydroxybutyrate, acetoacetate, and acetone), which induces faster weight loss than balanced low-caloric diets [69]. In a clinical trial, overweight patients (BMI > 25) with T2DM on a very low-carbohydrate Ketogenic Diet lost more weight and demonstrated improved glycemic control than a conventional, low-fat diabetes diet after a 32-week intervention [70]. Some previous studies testified that the very low-carbohydrate Ketogenic Diet can reverse the metabolic abnormalities of T2DM patients. Specifically, a Ketogenic Diet induces a rapid and sensible weight loss along with favorable biomarker changes, such as a reduction in serum HbA1c in T2DM patients. However, the substantial rise in the low-density lipoprotein cholesterol level has been a major concern among physicians [71]. No direct comparisons of the effects brought about by IF and the Ketogenic Diet have been performed yet, and the safety of a long-term Ketogenic Diet requires further investigation.

### 5.4. Comparison to Ramadan Intermittent Fasting

Ramadan Intermittent Fasting (RIF) is an old tradition among Muslims, taking place in the ninth month of the Islamic calendar. During the Ramadan month, all food and liquid is avoided from dawn to sunset among healthy adolescents and adults, and then food is consumed only after sunset. The studies on RIF have been increasing yearly; however, as a special form of fasting, its effects are more controversial. RIF has been demonstrated to exert a more marginal effect on weight loss. Faris et al. [72] reported in their study that the body weight of the participants was reduced by 1.2 kg on the average, in the absence of a significant body composition change after the Ramadan month. Meanwhile, a previous clinical trial showed that the serum total cholesterol, triglycerides, and HDL-cholesterol in Muslims were decreased significantly by RIF [72]. Interestingly and controversially, some studies observed weight gain [73], which might have resulted from the increased energy intake during the evening meal and changes in circadian rhythms. Previous evidence also suggested that RIF potentially increases cardiometabolic risk, which was proposed to be caused by the change in circadian rhythm with the mealtimes. For instant, Ahmed et al. [74] reported that the desynchrony of the circadian system gives rise to cardiometabolic disorders and eating at the wrong time leads to misalignment of the biological clock involved in the circadian system. Together with the minor effect on weight loss, this is not considered a feasible strategy to control weight and metabolism for the entire population.

### 5.5. Comparison to Christian Orthodox Fasting

Christian Orthodox Fasting (OF) is adopted and followed by a large proportion of the Orthodox population and assimilated into their daily eating patterns. As the Mediterranean Diet is indicated to be a healthy diet, Christian Orthodox Fasting incorporates the common characteristics of the Mediterranean Diet. Similar to the Mediterranean Diet, food and liquid are avoided from dawn to sunset during fast periods, and the fasting period and non-fasting period alternate during the calendar year; it also involves specific food choices. Fasters refrain from oil, meat, eggs, fish, and dairy products every Wednesday and Friday, and during major fasting periods [75].

To compare the late metabolic effects of TRF and OF, Karras et al. [10] conducted TRF (feeding in 8:00–16:00) and OF (total of 1200–1500 kilocalories per day for women and 1500–1800 kilocalories per day for men, fasting from dusk till dawn) among 45 obese participants for 7 weeks. It was found that both groups had a tendency towards weight loss (*p* < 0.001), which was mediated by the decreased energy intake. The OF group showed a downwards trend at the 7th week, although this was not significant at the 13th week (6 weeks after the end of the intervention).

Through a prospective, interventional, two-arm study including 97 premenopausal women for 7 weeks, it was also testified that OF might increase the adiponectin values, which has been reported to mediate glucose and lipid homeostasis and improve insulin sensitivity [11]. During the entire study period, an inverse correlation was found between adiponectin and waist circumference values.

Meanwhile, it was found that OF also induced a superior reduction in TC and atherogenic LDL-C concentrations compared to TRF in a small pilot study on 37 overweight adults, indicating that OF may have a better influence in reducing lipids [75]. The potential regulatory mechanism may be related to the decreased consumption of saturated fatty acids (SFAs), which results in an increase in LDL receptors and restricted energy intake. Moreover, OF also led to higher TG concentrations, probably due to the increased carbohydrate intake, which may lead to hypertriglyceridemia [10]. Similar to IF, the limited evidence indicates that more long-term and large-scale studies are required to delve into the differences in the mechanism and effects of OF and RIF.

## 6. Challenges

As reviewed above, multiple lines of evidence from animal studies and human trials have shown the promising effects of intermittent fasting on human health. With all the benefits, IF is gaining more attention and becoming a new strategy for weight loss among young people. Nevertheless, the risks and challenges should also be noted.

### 6.1. Appetite Change

Proper appetite is crucial to the health of living organisms [73]. The change, loss, or gain of appetite may be a concern that has to be dealt with during or after IF intervention. Hence, Beaulieu et al. [76] conducted a 12-week clinical trial on women with overweight and obesity, and assessed the subjective appetite reaction to an assured energy breakfast and eating behavior traits after the intervention of intermittent or continuous energy restriction. The result showed that the intervention of intermittent or continuous energy restriction did not lead to a compensatory increase in appetite or appetite loss, when weight loss controlled by intermittent or continuous energy restriction was not less than 5%. However, this conclusion was not in line with an Early Time-Restricted Feeding study. Eleven overweight adults participated in both Early Time-Restricted Feeding (eTRF) (eating from 8 a.m. to 2 p.m.) and a control schedule (eating from 8 a.m. to 8 p.m.) for 4 days each, and it was found that eTRF decreased mean ghrelin levels and the desire to eat among the participants [26]. Whether IF influences the appetite to facilitate weight loss still requires investigation through more rigorous studies.

### 6.2. Safety and Tolerability

The present evidence indicates that, regarding overweight and obese people, including adults and adolescents, IF is a feasible, effective, safe, and tolerable treatment for weight loss. Catenacci et al. [5] showed that, after an 8-week ADF intervention among 14 obese adults, no adverse reaction was observed in the contribution of ADF, with more than 90% of the participants completing the ADF process. Furthermore, in an IER intervention trial in obese adolescents, 30 adolescents were recruited and 21 completed the study, and it was concluded that IER was effective at reducing BMI and cardiometabolic risk and it was an acceptable treatment for obese adolescents [77]. Regardless, we should not overestimate the benefits and should maintain a skeptical and cautious attitude towards the current suggestion about the IF influence before further investigation is carried out to confirm that there is no long-term harm for human health.

## 7. Conclusions

The global epidemic of obesity and diabetes demands a feasible and effective intervention in a healthcare-manageable method. Short-term IF presents promising efficacy for weight loss in metabolic disorder patients, and the positive influences on weight loss and metabolism benefits for obesity and T2DM patients have been well documented. By improving glucose homeostasis, IF can potentially become an adjuvant treatment for T2DM. Moreover, the other benefits of IF among obesity patients, such as the recovery of muscle function and reduction of cardiometabolic risk, may provide more fundamental support for IF to become a promising approach in rehabilitation treatment for chronic non-communicable diseases.

Nevertheless, T2DM patients should be more cautious when being treated with IF, ensuring the involvement of their physicians to ensure that the intervals of not eating are tolerated as the potential risk is raised [78]. IF may also have a beneficial effect on weight loss among young people, similar to the behavior intervention in the clinical treatment of obesity. However, Clayton et al. [79] showed that 24 h substantial energy restriction has an acute impact on indices of insulin sensitivity in lean males. Therefore, dieters are also advised to be guided by dietitians or physicians to avoid potential side effects when they participate in IF.

In conclusion, the current studies that focus on animal experiments, short-time interventions, and small-scale human trials have provided convincing evidence to prove that short-term IF is healthy and safe. More large-scale cohort studies need to be performed to confirm the long-term benefits and potential harms amongst patients, particularly for humans who are incapable of adipose tissue browning or are under insulin resistance (e.g., ageing and diabetes), and the efficacy amongst normal-weight subjects still warrants further investigation, which will provide deeper insights to potentially determine the appropriate and optimal prescription for long-term successful treatments in metabolic diseases and general personal healthcare management.

## Figures and Tables

**Table 1:** The characteristics of the most common forms of intermittent fasting.

**Features**	**Intermittent Energy Restriction**	**Time-Restricted Fasting**
Logic involved	Reduction compensatory metabolic responses	Circadian rhythms
Main principle of weight loss	The induction of energy intakes	The induction of energy intake time
Fasting period	A set of days, normally in a week	No more than 24 h
Intakes in fasting period	Strictly controlled energy intake	Only water or nothing
Common patterns	Twice-per-week fast, every-other-day fast (ADF)	16:8 diet, 15:9 diet

**Table 2:** The impact of intermittent fasting on obesity (animal studies).

First Author, Year	*n*	Subject	Regimen	Duration	Key Results
Liu, 2019 [31]	48	10-week-old male C57BL/6J mice	IF (24-h fast on 3 non-consecutive days per week)	16 weeks (6-week diet and 6-week follow-up)	16 weeks (6-week diet and 6-week follow-up)
Villanueva, 2019 [32]	100–150 per group	Drosophila melanogaster (fruit fly)	TRF (TRF flies only had access to water at 12-h night) 3 weeks	3 weeks	Flight index ↑ (compared to control group, *p* < 0.05); climbing performance ↑ (compared to control group, *p* < 0.05)
Kim, 2019 [34]	10–16	7-week-old male C57BL/6J mice	2:1 Isocaloric IF Regimen	16 weeks	Weight ↓ (*p* < 0.05); higher insulin sensitivity under HFD (compared to control group)
Olsen, 2017 [35]	50	4-week-old male Sprague-Dawley rats	TRF (feeding in 21:00–6:00)	9 weeks	Weight ↓ (*p* < 0.05)
Swoap, 2019 [36]	34	13-week-old female ob/ob mice and female ob/+ littermate mice	ADF	19 days	Weight did not change
Kim, 2019 [37]	6–11 per group	6-week-old leptin-deficient ob/ob male mice	2:1 IF (2 days of feeding—1 day of fasting)	16 weeks	Weight gain did not reduce (compared to control group): Glucose excursions ↓ (*p* < 0.05)
Dedual, 2019 [44]	3–5 per group	12-week-old male C57BL/6J wild-type littermate mice	IF (fasted for eight hours during their inactive phase)	4 days	mRNA expression of enzymes involved in lipogenesis ↑ (*p* < 0.05); hepatic triglyceride levels ↑ (*p* < 0.05); various triglyceride and diacylglycerol fractions in the liver ↑ (*p* < 0.05)
Li, 2017 [52]	7–10 mice per group	6-week-old male C57BL/6N mice	Every-other-day fasting (fed with alternating 24 h periods)	30 days	The ratio of *Firmicutes: Bacteroidetes* increased from 3.4 in AL mice to 8.9 in EODF mice; intestine length ↑ (*p* < 0.05)

**Table 3:** The impact of intermittent fasting on obesity (clinical studies).

First Author, Year	*n*	Subject	Regimen	Duration	Key Results
Catenacci, 2016 [5]	29	Obese adults (aged 18–55 years, BMI 30 kg/m^2^)	Zero-calorie ADF; CR (−400 kcal/day)	32 weeks (8-month diet and 24-month follow-up)	Both weight ↓ (CR −7.1 ± 1.0 kg, ADF −8.2 ± 0.9 kg, *p* < 0.05)
Currenti, 2021 [12]	1936	Adult (>18 years old)	TRF (8 h feeding window/10 h fasting window)	6 months	Being obese ↓ [TRF-10, OR = 0.05, 95% CI: (0.01, 0.07); TRF-8, OR = 0.08, 95% CI: (0.04, 0.15)];hypertension [TRF-10, OR = 0.24, 95% CI: (0.13, 0.45); TRF-8, OR = 0.33, 95% CI: (0.17, 0.60)]
Antoni, 2016 [14]	10 (3 female)	Healthy, overweight/obese participants (aged 36 ± 5 years; BMI 29.0 ± 1.1 kg/m^2^)	IER (total/100% ER and partial/75%ER)	3 days	Both fasting plasma glucose ↓ (*p* < 0.05); TAG ↓ (*p* < 0.05); postprandial glucose time to peak was significantly delayed
Kim, 2020 [33]	43	Individuals with central obesity (aged 35–75 years)	IER (5:2 pattern) and continuous energy restriction	4 weeks	Both pattern separation ↑ (*p* = 0.0005); only the IER group recognition memory ↓ (*p* < 0.05)
Dorothea, 2019 [42]	40 (31 females)	Abdominally obese participants (waist-to-height ratio, WHtR ≥ 0.5, aged 49.1 ± 12.4 years)	TRF (limit the daily period of food intake to 8–9 h and extend nightly fasting period to 15–16 h)	3 months	Waist circumference ↓ (−5.3 ± 3.1 cm, *p* < 0.001); HbA1c ↓ −1.4 ± 3.5 mmol/mol (*p* = 0.003)
Sundfør, 2018 [43]	112 (50% males and 50% females)	Obese participants (aged 21–70 years, BMI 30–45 kg/m^2^)	IER and continuous energy restriction	12 months (6-month diet and 6 month maintenance phase)	Both weight ↓ (*p* < 0.05); waist circumference ↓ (*p* < 0.05); triglycerides ↓ (*p* < 0.05); HDL-cholesterol ↑ (*p* < 0.05)
Jones, 2020 [48]	16 (all males)	Healthy males (aged 23 ± 1 years; BMI 24.0 ± 0.6 kg/m^2^)	eTRF (daily energy intake was restricted to between 08:00 and 16:00)	2 weeks	Whole-body insulin sensitivity ↑ (*p* < 0.05); skeletal muscle uptakeof glucose ↑ (*p* < 0.05)
Gabel, 2020 [51]	14	Adults, obesity	TRF (8 h feeding window/16 h fasting window)	12 weeks	Weight ↓ (−2 ± 1 kg, *p* < 0.05); the abundance of Firmicutes, Bacteroidetes, or any other phyla did not have significant alternation
Bowen, 2018, [53]	136	Adult, obese (aged 40 ± 8 years, BMI 36 ± 6 kg/m^2^)	ADF + daily energy restriction/DER (alternated between the DER, a modified fasting regimen, and one day per week to eat ad libitum)	24 weeks (16-month diet and 8-month maintenance phase)	Fasting LDL-cholesterol, triglycerides, insulin, hsCRP, glucose, and blood pressure all improved (*p* < 0.05)
Trepanowski, 2017 [54]	100 (14 males and 86 females)	Adult (aged 44 ± 11 years; BMI 25.0–39.9 kg/m^2^)	ADF + daily calorie restriction	12 months (6-month diet and 6-month follow-up)	Weight ↓ (both group–6.0%, *p* < 0.05) ↓ (insulin, glucose) compared to control group

**Table 4:** The impact of intermittent fasting on diabetes.

First Author, Year	*n*	Subject	Regimen	Duration	Key Results
Corley, 2018 [13]	37	Adults [BMI of 30–45 kg/m^2^, type 2 diabetes treated with metformin and/or hypoglycemic medications and a HbA1c concentration of 50–86 mmol/mol (6.7–10%)]	IER (2092–2510 kJ diet on 2 days per week)	12 weeks	The rate of hypoglycemia ↑ (*p* < 0.05)
Hutchison, 2019 [22]	15 (all men)	Adults (age 55 ± 3 years, BMI 33.9 ± 0.8 kg/m^2^)	TRFe (8 a.m. to 5 p.m.) and TRFd (12 p.m. to 9 p.m.)	7 days	Glucose incremental area ↓ (*p* = 0.001); fasting triglycerides ↓ (*p* = 0.003); mean fasting glucose ↓ in TRFe (*p* = 0.02), but not TRFd (*p* = 0.17)
Carter, 2019 [50]	131	Adults, type 2 diabetes	IER (2100–2500 kJ diet 2 non-consecutive days/weekand their usual diet for 5 days/week) and continuous energy restriction (5000–6300 kJ diet for 7 days/week)	24 months (12-month diet and 12-month follow-up)	Both HbA1c level↑ (*p* < 0.05); weight ↓ (3.9 ± 1.1 kg, *p* < 0.001)
Drinda, 2019 [57]	697	Adults (age ≥ 18 years; BMI ≥ 19 kg/m^2^, 38 with T2DM)	Low-calorie transition day (600 kcal/day mono-diet)	Adapted to the individual therapeutic goal	BMI ↓ (−1.51 ± 0.82 kg/m^2^, *p* < 0.05); FLI↓(−14.02 ± 11.67; *p* < 0.0001)

## Data Availability

Not applicable.

## References

[1] Jaacks J.M., Vandevijvere S., Pan A., McGowan C.J., Wallace C., Imamura F., Mozaffarian D., Swinburn B., Ezzati M. (2019). The obesity transition: Stages of the global epidemic. Lancet Diabetes Endocrinol..

[2] WHO The Global Health Observatory [EB/OL]. https://www.who.int/data/gho/data/themes/theme-details/GHO/body-mass-index-.

[3] Chobot A., Górowska-Kowolik K., Sokołowska M., Jarosz-Chobot P. (2018). Obesity and diabetes-Not only a simple link between two epidemics. Diabetes/Metab. Res. Rev..

[4] Madeo F., Carmona-Gutierrez D., Hofer S.J., Kroemer G. (2019). Caloric restriction mimetics against age-associated disease: Targets, mechanisms, and therapeutic potential. Cell Metab..

[5] Catenacci V.A., Pan Z., Ostendorf D., Brannon S., Gozansky W.S., Mattson M.P., Martin B., MacLean P.S., Melanson E.L., Troy Donahoo W. (2016). A randomized pilot study comparing zero-calorie alternate-day fasting to daily caloric restriction in adults with obesity. Obesity.

[6] Hatori M., Vollmers C., Zarrinarp A., DiTacchio L., Bushong E.A., Gill S., Leblanc M., Chaix A., Joens M., Fitzpatrick J.A.J. (2012). Time-restricted feeding without reducing caloric intake prevents metabolic diseases in mice fed a high-fat diet. Cell Metab..

[7] Liu H., Javaheri A., Godar R.J., Murphy J., Ma X., Rohatgi N., Mahadevan J., Hyrc K., Saftig P., Marshall C. (2017). Intermittent fasting preserves beta-cell mass in obesity-induced diabetes via the autophagy-lysosome pathway. Autophagy.

[8] Tallis J., James R.S., Seebacher F. (2018). The effects of obesity on skeletal muscle contractile function. J. Exp. Biol..

[9] Grosso G. (2021). Intermittent fasting: Promising premises or broken promises?. Int. J. Food Sci. Nutr..

[10] Karras S.N., Koufakis T., Adamidou L., Polyzos S.A., Karalazou P., Thisiadou K., Zebekakis P., Makedou K., Kotsa K. (2021). Similar late effects of a 7-week orthodox religious fasting and a time restricted eating pattern on anthropometric and metabolic profiles of overweight adults. Int. J. Food Sci. Nutr..

[11] Karras S.N., Koufakis T., Adamidou L., Dimakopoulos J., Karalazou P., Thisiadou K., Makedou K., Zebekakis P., Kotsa K. (2021). Implementation of Christian Orthodox fasting improves plasma adiponectin concentrations compared with time-restricted eating in overweight premenopausal women. Int. J. Food Sci. Nutr..

[12] Currenti W., Buscemi S., Cincione R.I., Cernigliaro A., Godos J., Grosso G., Galvano F. (2021). Time-restricted feeding and metabolic outcomes in a cohort of Italian adults. Nutrients.

[13] Corley B.T., Carroll R.W., Hall R.M., Weatherall M., Parry-Strong A., Krebs J.D. (2018). Intermittent fasting in Type 2 diabetes mellitus and the risk of hypoglycaemia: A randomized controlled trial. Diabet. Med..

[14] Antoni R., Johnston K.L., Collins A.L., Robertson M.D. (2016). Investigation into the acute effects of total and partial energy restriction on postprandial metabolism among overweight/obese participants. Br. J. Nutr..

[15] Gabel K., Hoddy K.K., Varady K.A. (2019). Safety of 8-h time restricted feeding in adults with obesity. Appl. Physiol. Nutr. Metab..

[16] Byrne N.M., Sainsbury A., King N.A., Hills A.P., Wood R.E. (2018). Intermittent energy restriction improves weight loss efficiency in obese men: The MATADOR study. Int. J. Obes..

[17] Davis C.S., Clarke R.E., Coulter S.N., Rounsefell K.N., Walker R.E., Rauch C.E., Huggins C.E., Ryan L. (2016). Intermittent energy restriction and weight loss: A systematic review. Eur. J. Clin. Nutr..

[18] Lundell L.S., Parr E.B., Devlin B.L., Ingerslev L.R., Altıntaş A., Sato S., Sassone-Corsi P., Barrès R., Zierath J.R., Hawley J.A. (2020). Time-restricted feeding alters lipid and amino acid metabolite rhythmicity without perturbing clock gene expression. Nat. Commun..

[19] Rynders C.A., Thomas E.A., Zaman A., Pan Z., Catenacci V.A., Melanson E.L. (2019). Effectiveness of intermittent fasting and time-restricted feeding compared to continuous energy restriction for weight loss. Nutrients.

[20] Longo V.D., Panda S. (2016). Fasting, circadian rhythms, and time-restricted feeding in healthy lifespan. Cell Metab..

[21] Dong T.A., Sandesara P.B., Dhindsa D.S., Mehta A., Arneson L.C., Dollar A.L., Taub P.R., Sperling L.S. (2020). Intermittent fasting: A heart healthy dietary pattern?. Am. J. Med..

[22] Hutchison A.T., Regmi P., Manoogian E.N.C., Fleischer J.G., Wittert G.A., Panda S., Heilbronn L.K. (2019). Time-restricted feeding improves glucose tolerance in men at risk for type 2 diabetes: A randomized crossover trial. Obesity.

[23] Nencioni A., Caffa I., Cortellino S., Longo V.D. (2018). Fasting and cancer: Molecular mechanisms and clinical application. Nat. Rev. Cancer.

[24] Anton S.D., Moehl K., Donahoo W.T., Marosi K., Lee S.A., Mainous A.G., Leeuwenburgh C., Mattson P. (2018). Flipping the metabolic switch: Understanding and applying the health benefits of fasting. Obesity.

[25] Li G., Xie C., Lu S., Ma Y., Brocker C.N., Yan T., Krausz K.W., Xiang R., Gavrilova O., Patterson A.D. (2017). Intermittent fasting promotes white adipose browning and decreases obesity by shaping the gut microbiota. Cell Metabol..

[26] Ravussin E., Beyl R.A., Poggiogalle E., Hsia D.S., Peterson C.M. (2019). Early time-restricted feeding reduces appetite and increases fat oxidation but does not affect energy expenditure in humans. Obesity.

[27] Liu B., Du Y., Wu Y., Snetselaar L.G., Wallace R.B., Bao W. (2021). Trends in obesity and adiposity measures by race or ethnicity among adults in the United States 2011–18: Population based study. BMJ.

[28] Fitzgerald F.T. (1981). The problem of obesity. Annu. Rev. Med..

[29] Puhl R., Heuer C.A. (2010). Obesity stigma: Important considerations for public health. Am. J. Public Health.

[30] Park J., Seo Y.G., Paek Y.J., Song H.J., Park K.H., Noh H.M. (2020). Effect of alternate-day fasting on obesity and cardiometabolic risk: A systematic review and meta-analysis. Metabolism.

[31] Liu B., Page A.J., Hatzinikolas G., Chen M., Wittert G.A., Heilbronn L.K. (2019). Intermittent fasting improves glucose tolerance and promotes adipose tissue remodeling in male mice fed a high-fat diet. Endocrinology.

[32] Villanueva J.E., Livelo C., Trujillo A.S., Chandran S., Woodworth B., Andrade L., Le H.D., Manor U., Panda S., Melkani G.C. (2019). Time-restricted feeding restores muscle function in Drosophila models of obesity and circadian-rhythm disruption. Nat. Commun..

[33] Kim C., Pinto A.M., Bordoli C., Buckner L.P., Kaplan P.C., Del Arenal I.M., Jeffcock E.J., Hall W.L., Thuret S. (2020). Energy restriction enhances adult hippocampal neurogenesis-associated memory after four weeks in an adult human population with central obesity; a randomized controlled trial. Nutrients.

[34] Kim R.Y., Lee J.H., Oh Y., Sung H.K., Kim K.H. (2019). Assessment of the metabolic effects of isocaloric 2:1 intermittent fasting in mice. J. Vis. Exp..

[35] Olsen M.K., Choi M.H., Kulseng B., Zhao C.M., Chen D. (2017). Time-restricted feeding on weekdays restricts weight gain: A study using rat models of high-fat diet-induced obesity. Physiol. Behav..

[36] Swoap S.J., Bingaman M.J., Hult E.M., Sandstrom N.J. (2019). Alternate-day feeding leads to improved glucose regulation on fasting days without significant weight loss in genetically obese mice. Am. J. Physiol. Regul. Integr. Comp. Physiol..

[37] Kim Y.H., Lee J.H., Yeung J.L., Das E., Kim R.Y., Jiang Y., Moon J.H., Jeong H., Thakkar N., Son J.E. (2019). Thermogenesis-independent metabolic benefits conferred by isocaloric intermittent fasting in ob/ob mice. Sci. Rep..

[38] Lanier V., Gillespie C., Leffers M., Daley-Brown D., Milner J., Lipsey C., Webb N., Anderson L.M., Newman G., Waltenberger J. (2016). Leptin-induced transphosphorylation of vascular endothelial growth factor receptor increases Notch and stimulates endothelial cell angiogenic transformation. Int. J. Biochem. Cell Biol..

[39] Yang W.H., Chen J.C., Hus K.H., Lin C.Y., Wang S.W., Wang S.Y., Chang Y.S., Tang C.H. (2014). Leptin increases VEGF expression and enhances angiogenesis in human chondrosarcoma cells. Biochim. Biophys. Acta..

[40] Park H.Y., Kwon H.M., Lim J.M., Hong B.K., Lee J.Y., Park B.E., Jang Y., Cho S.Y., Kim H.S. (2001). Potential role of leptin in angiogenesis: Leptin induces endothelial cell proliferation and expression of matrix metalloproteinases in vivo and in vitro. Exp. Mol. Med..

[41] Silveira E.A., Vaseghi G., Santos A.S., Kliemann N., Masoudkabir F., Noll M., Mohammadifard N., Sarrafzadegan N., Oliveira C.D. (2020). Visceral obesity and its shared role in cancer and cardiovascular disease: A scoping review of the pathophysiology and pharmacological treatments. Int. J. Mol. Sci..

[42] Dorothea K., Petra C., Markus G., Tibor K. (2019). Adherence to time-restricted feeding and impact on abdominal obesity in primary care patients: Results of a pilot study in a pre–post design. Nutrients.

[43] Sundfør T.M., Svendsen M., Tonstad S. (2018). Effect of intermittent versus continuous energy restriction on weight loss, maintenance and cardiometabolic risk: A randomized 1-year trial. Nutr. Metab. Cardiovasc. Dis..

[44] Dedual M.A., Wueest S., Borsigova M., Konrad D. (2019). Intermittent fasting improves metabolic flexibility in short-term high-fat diet-fed mice. Am. J. Physiol. Endocrinol. Metab..

[45] Chaix A., Lin T., Le H.D., Chang M.W., Panda S. (2019). Time-Restricted feeding prevents obesity and metabolic syndrome in mice lacking a circadian clock. Cell Metab..

[46] Chaix A., Zarrinpar A., Miu P., Panda S. (2014). Time-restricted feeding is a preventative and therapeutic intervention against diverse nutritional challenges. Cell Metab..

[47] Woodie L.N., Luo Y., Wayne M.J., Graff E.C., Ahmed B., O’Neill A.M., Greene M.W. (2018). Restricted feeding for 9h in the active period partially abrogates the detrimental metabolic effects of a Western diet with liquid sugar consumption in mice. Metab. Clin. Exp..

[48] Jones R., Pabla P., Mallinson J., Nixon A., Taylor T., Bennett A., Tsintzas K. (2020). Two weeks of early time-restricted feeding (eTRF) improves skeletal muscle insulin and anabolic sensitivity in healthy men. Am. J. Clin. Nutr..

[49] Magne F., Gotteland M., Gauthier L., Zazueta A., Pesoa S., Navarrete P., Balamurugan R. (2020). The firmicutes/bacteroidetes ratio: A relevant marker of gut dysbiosis in obese patients?. Nutrients.

[50] Secor S.M., Carey H.V. (2016). Integrative physiology of fasting. Compr. Physiol..

[51] Gabel K., Marcell J., Cares K., Kalam F., Cienfuegos S., Ezpeleta M., Varady K. (2020). A Effect of time restricted feeding on the gut microbiome in adults with obesity: A pilot study. Nutr. Health..

[52] Galicia-Garcia U., Benito-Vicente A., Jebari S., Larrea-Sebal A., Siddiqi H., Uribe K.B., Ostolaza H., Martín C. (2020). Pathophysiology of type 2 diabetes mellitus. Int. J. Mol. Sci..

[53] Bowen J., Brindal E., James-Martin G., Noakes M. (2018). Randomized trial of a high protein, partial meal replacement program with or without alternate day fasting: Similar effects on weight loss, retention status, nutritional, metabolic, and behavioral outcomes. Nutrients.

[54] Trepanowski J.F., Kroeger C.M., Barnosky A., Bhutani S., Hoddy K.K., Gabel K., Freels S., Rigdon J., Rood J., Ravussin E. (2018). Effect of alternate-day fasting on weight loss, weight maintenance, and cardioprotection among metabolically healthy obese adults: A randomized clinical trial. JAMA Int. Med..

[55] Carter S., Clifton P.M., Keogh J.B. (2019). The effect of intermittent compared with continuous energy restriction on glycaemic control in patients with type 2 diabetes: 24-month follow-up of a randomised noninferiority trial. Diabetes Res. Clin. Pract..

[56] Toledo F.W.D., Buchinger A., Burggrabe H., Hölz G., Kuhn C., Lischka E., Lischka N., Lützner H., May W., Ritzmann-Widderich M. (2013). Fasting therapy–An expert panel update of the 2002 consensus guidelines. Forsch. Komplement..

[57] Drinda S., Grundler F., Neumann T., Lehmann T., Steckhan N., Michalsen A., De Toledo F.W. (2019). Effects of periodic fasting on fatty liver index—A prospective observational study. Nutrients.

[58] Wu H., Tremaroli V., Schmidt C., Lundqvist A., Olsson L.M., Krämer M., Gummesson A., Perkins R., Bergström G., Bäckhed F. (2020). The gut microbiota in prediabetes and diabetes: A population-based cross-sectional study. Cell Metab..

[59] Beli E., Yan Y., Moldovan L., Vieira C.P., Gao R., Duan Y., Prasad R., Bhatwadekar A., White F.A., Townsend S.D. (2018). Restructuring of the gut microbiome by intermittent fasting prevents retinopathy and prolongs survival in db/db mice. Diabetes.

[60] Weir H.J., Yao P., Huynh F.K., Escoubas C.C., Goncalves R.L., Burkewitz K., Laboy R., Hirschey M.D., Mair W.B. (2017). Dietary restriction and AMPK increase lifespan via mitochondrial network and peroxisome remodeling. Cell Metab..

[61] Gabel K., Kroeger C.M., Trepanowski J.F., Hoddy K.K., Cienfuegos S., Kalam F., Varady K.A. (2019). Differential effects of alternate-day fasting versus daily calorie restriction on insulin resistance. Obesity.

[62] Smith N.J., Caldwell J.L., van der Merwe M., Sharma S., Butawan M., Puppa M., Bloomer R.J. (2019). A comparison of dietary and caloric restriction models on body composition, physical performance, and metabolic health in young mice. Nutrients.

[63] Mentella M.C., Scaldaferri F., Ricci C., Gasbarrini A., Miggiano G.A.D. (2019). Cancer and Mediterranean Diet: A review. Nutrients.

[64] Reynolds A., Mann J., Cummings J., Winter N., Mete E., Te L. (2019). Morenga Carbohydrate quality and human health: A series of systematic reviews and meta-analyses. Lancet.

[65] Jospe M.R., Roy M., Brown R.C., Haszard J.J., Meredith-Jones K., Fangupo L.J., Osborne H., Fleming E.A., Taylor R.W. (2020). Intermittent fasting, Paleolithic, or Mediterranean diets in the real world: Exploratory secondary analyses of a weight-loss trial that included choice of diet and exercise. Am. J. Clin. Nutr..

[66] Yannakoulia M., Kontogianni M., Scarmeas N. (2015). Cognitive health and Mediterranean diet: Just diet or lifestyle pattern?. Ageing Res. Rev..

[67] Sofi F., Cesari F., Abbate R., Gensini G.F., Casini A. (2008). Adherence to Mediterranean diet and health status: Meta-analysis. BMJ.

[68] Boison D. (2017). New insights into the mechanisms of the ketogenic diet. Curr. Opin. Neurol..

[69] D’Abbondanza M., Ministrini S., Pucci G., Migliola E.N., Martorelli E.E., Gandolfo V., Siepi D., Lupattelli G., Vaudo G. (2020). Very low-carbohydrate ketogenic diet for the treatment of severe obesity and associated non-alcoholic fatty liver disease: The role of sex differences. Nutrients.

[70] Saslow L.R., Mason A.E., Kim S., Goldman V., Ploutz-Snyder R., Bayandorian H., Daubenmier J., Hecht F.M., Moskowitz J.T. (2017). An online intervention comparing a very low-carbohydrate ketogenic diet and lifestyle recommendations versus a plate method diet in overweight individuals with type 2 diabetes: A randomized controlled trial. J. Med. Internet Res..

[71] O’Neill B., Raggi P. (2020). The ketogenic diet: Pros and cons. Atherosclerosis.

[72] Faris M., Madkour M.I., Obaideen A.K., Dalah E.Z., Hasan H.A., Radwan H., Jahrami H.A., Hamdy O., Mohammad M.G. (2019). Effect of Ramadan diurnal fasting on visceral adiposity and serum adipokines in overweight and obese individuals. Diabetes Res. Clin. Pract..

[73] Lessan N., Ali T. (2019). Energy Metabolism and Intermittent Fasting: The Ramadan Perspective. Nutrients.

[74] BaHammam A., Almeneessier A.S. (2020). Recent Evidence on the Impact of Ramadan Diurnal Intermittent Fasting, Mealtime, and Circadian Rhythm on Cardiometabolic Risk: A Review. Front. Nutr..

[75] Karras S.N., Koufakis T., Adamidou L., Antonopoulou V., Karalazou P., Thisiadou K., Mitrofanova E., Mulrooney H., Petróczi A., Zebekakis P. (2021). Effects of orthodox religious fasting versus combined energy and time restricted eating on body weight, lipid concentrations and glycaemic profile. Int. J. Food Sci. Nutr..

[76] Beaulieu K., Casanova N., Oustric P., Turicchi J., Gibbons C., Hopkins M., Varady K., Blundell J., Finlayson G. (2020). Matched weight loss through intermittent or continuous energy restriction does not lead to compensatory increases in appetite and eating behavior in a randomized controlled trial in women with overweight and obesity. Nutrition.

[77] Jebeile H., Gow M.L., Lister N.B., Haghighi M.M., Ayer J., Cowell C.T., Baur L.A., Garnett S.P. (2019). Intermittent energy restriction is a feasible, effective, and acceptable intervention to treat adolescents with obesity. Nutrition.

[78] Horne B.D., Grajower M.M., Anderson J.L. (2020). Limited evidence for the health effects and safety of intermittent fasting among patients with type 2 diabetes. JAMA.

[79] Clayton D.J., Biddle J., Maher T., Funnell M.P., Sargeant J.A., King J.A., Hulston C.J., Stensel D.J., James L.J. (2018). 24-h severe energy restriction impairs postprandial glycaemic control in young, lean males. Br. J. Nutr..

